# Sesquiterpene Biosynthetic Gene *vir4* from *Trichoderma virens* Enhances Direct Herbivore Resistance while Maintaining Indirect Defense

**DOI:** 10.1007/s10886-025-01681-4

**Published:** 2026-01-13

**Authors:** Noor Agha Nawakht, Artemio Mendoza-Mendoza, Michael Rostás

**Affiliations:** 1 Agricultural Entomology, Department of Crop Sciences, Georg-August University, Grisebachstraße 6, Göttingen, 37077 Germany; 2 Agriculture and Life Sciences Faculty, Lincoln University, Lincoln, New Zealand; 3 Faculty of Agriculture, Nangarhar University, Nangarhar, Afghanistan

**Keywords:** Trichoderma virens, Helicoverpa armigera, Macrolophus pygmaeus, Maize, Tri-trophic interactions, GC-MS, HIPVs, Defense priming

## Abstract

*Trichoderma* species are widely used as root-colonizing biocontrol agents that enhance plant resistance to biotic and abiotic stresses while promoting growth. These fungi produce diverse volatile and non-volatile metabolites that mediate interactions with plants. *Trichoderma* can influence both direct and indirect plant defenses, including the release of herbivore-induced plant volatiles (HIPVs) that attract natural enemies of herbivores. In this study, we examined the effects of *T. virens* and its *vir4* gene *(*regulating terpenoid synthesis) knockout-mutant on maize (*Zea mays*), the herbivore *Helicoverpa armigera*, and its predator *Macrolophus pygmaeus*. Previous research has shown that *T. virens* differentially modulates maize root gene expression and specialized metabolite concentrations. Here, we found that caterpillars feeding on maize seedlings colonized by wild-type *T. virens* gained significantly less weight than those feeding on maize colonized by the *vir4* knockout mutant or uncolonized plants, suggesting that the *vir4* gene cluster contributes to herbivore resistance. Although fungal colonization led to moderate changes in HIPV composition, total volatile emissions remained unchanged. In Y-tube assays, *M. pygmaeus* preferred caterpillar-infested maize over healthy plants, but fungal colonization did not significantly affect predator behavior. Our findings demonstrate that *T. virens* enhances direct plant defense against herbivores while maintaining indirect defense through a mechanism regulated by terpenoid synthesis depending on *vir4* gene. Further research is needed to elucidate the metabolic changes in maize induced by *T. virens* that contribute to reduced herbivore performance.

**Supplementary Information:**

The online version contains supplementary material available at 10.1007/s10886-025-01681-4.

## Introduction

The genus *Trichoderma* (teleomorph Hypocrea) is a globally distributed soil-borne fungus that thrives in diverse habitats (Woo et al. 2022). It plays a key role in sustainable agriculture, acting as a biopesticide, bio-stimulant, and plant growth promoter while enhancing plant defenses (Harman et al. 2004; Woo et al. 2022). As an opportunistic symbiont, *Trichoderma* colonizes the rhizosphere and the apoplast of plant roots, inhabiting intercellular spaces and outer cell layers (Harman et al. 2004; Hermosa et al. 2012; Brotman et al. 2013; Nogueira-Lopez et al. 2018). Traditionally, its biocontrol activity was attributed to mycoparasitism, antibiosis, and competition for space and resources (Harman 2005; Vinale et al. 2008). However, recent studies highlight its role in inducing systemic and localized plant defense responses (Nawrocka 2013; Poveda 2021; Monte 2023). *Trichoderma* spp. produce a diverse array of volatile and non-volatile secondary metabolites that contribute to their bioactivity and defense-inducing properties in host plants (Hermosa et al. 2014; Jiménez-Bremont et al. 2024; Reino et al. 2007; Salwan et al. 2019; Vinale & Sivasithamparam. 2020; Zeilinger et al. 2016).

Colonization of maize roots by *Trichoderma* spp. can significantly alter the host plant’s metabolome (Vinale et al. 2008; Vinci et al. 2018), proteome, and transcriptome (Harman 2005; Marra et al. 2006; Alfano et al. 2007; Shoresh and Harman 2008; Nogueira-Lopez et al. 2018). Schweiger et al. (2021) demonstrated that the wild-type *T. virens* strain Gv29.8 and its *Δvir4* mutant, which is deficient in sesquiterpene biosynthesis, differentially modulate maize root gene expression and metabolic composition. Their study revealed that *T. virens* colonization profoundly affects several metabolites derived from the shikimate pathway, including flavonoids and other compounds, in a genotype-dependent manner. These metabolic changes may influence not only belowground plant-insect interactions but also aboveground interactions, as *Trichoderma* spp. can induce systemic resistance in leaves, potentially triggering cascading effects across trophic levels. Several studies have reported *Trichoderma*-induced resistance against herbivorous insects from different feeding guilds, including lepidopterans (Contreras-Cornejo et al. 2018; Coppola et al. 2019a; Macías-Rodríguez et al. 2020). For example, *T. atroviride* reduced aphid and caterpillar fitness in maize and tomato by modulating plant defense mechanisms (Coppola et al. 2019b). Similarly, *T. harzianum* negatively affected *Nezara viridula* feeding behavior in tomato by enhancing direct defense responses (Alinc et al. 2021).

Plants communicate with their environment through volatile organic compounds (VOCs), which can mediate both above- and belowground interactions (Massalha et al. 2017; Thompson et al. 2023). Insect feeding, in particular, triggers the release of herbivore-induced plant volatiles (HIPVs), which play a critical role in plant defense by attracting natural enemies of herbivores (Aartsma et al. 2017; Hilker & Meiners. 2006; Dicke et al. 2009). Microbial colonization, including beneficial fungi, can influence the emission of HIPVs by modifying both the composition and quantity of emitted volatiles (Coppola et al. 2017; Alinc et al. 2024). These changes, in turn, may affect the behavior and effectiveness of natural enemies in locating their herbivorous prey (Holopainen & Gershenzon. 2010; Pangesti et al. 2013; Pineda et al. 2010; Fernandez-Conradi et al. 2018; Schausberger et al. 2012). For instance, *Trichoderma harzianum* T22 was shown to enhance VOC-mediated defenses by increasing the attraction of aphid parasitoids in tomato (Coppola et al. 2017). Furthermore, *T. harzianum* and *T. atroviride* strain IMI 206,040 improve maize tolerance to herbivores by modifying the emission of volatile terpenes that attract predators (Contreras-Cornejo et al. 2018, 2021). Similarly, *T. harzianum* primes tomato plants to emit VOCs that attract parasitoids of aphids and stink bugs (Coppola et al. 2017; Alinc et al. 2024).

*Helicoverpa armigera* (Lepidoptera: Noctuidae) is a polyphagous caterpillar that infests economically important crops, including maize (*Zea mays* L.). Its high fecundity, broad host range, and facultative diapause contribute to its status as a major pest. The zoophytophagous mirid bug *M. pygmaeus* (Hemiptera: Miridae) is an efficient predator of various herbivorous pest insect including *H. armigera* and is attracted to HIPVs emitted by crops such as tomato and tobacco (Maselou et al. 2019; Ingegno et al. 2016; Urbaneja et al. 2009; Ebrahimi et al. 2019). However, no studies have examined *M. pygmaeus* attraction to maize HIPVs particularly in the presence of *T. virens* strains as endophytic root colonizers.

Since *T. virens* and its *vir4* knockout mutant influence maize root metabolic profile (Schweiger et al. 2021), we hypothesized that these changes could extend systemically to higher trophic levels, affecting herbivores and their natural enemies aboveground. Therefore, this study aimed to investigate: (i) how maize root colonization by both *T. virens* genotypes affects *H. armigera* survival and weight gain, (ii) whether fungal colonization alters maize VOC composition or quantity and (iii) the consequences of these changes for *M. pygmaeus* foraging behavior within this multitrophic system.

## Materials and methods

### Insects rearing

Eggs of *H. armigera* were supplied by Bayer AG Crop Sciences (Monheim, Germany), and the larvae were reared on an artificial diet. Eggs were placed in plastic containers with slices of the diet and kept in an environmental chamber at 23 °C; 65% RH; 14:10 h light: dark to induce hatching. After hatching, the neonates were reared on artificial diet until they reached the desired developmental stage, after which they were used in experiments. Adults of *M. pygmaeus* were obtained from Katz Biotech AG (Germany), along with *Sitotroga* eggs as supplementary food. A colony was established in BugDorm insect-rearing cages (BugDorm^®^, MegaView Science Co., Ltd.Taiwan). Potato tubers were placed on moistened soil in a tray inside the cages, and *Sitotroga* eggs were provided in a small Petri dish. Male and female adults of *M. pygmaeus* were released into the cage for mating and reproduction. Emerging adults were continuously transferred to new cages, maintained at ambient room temperature.

### Fungal culture and inoculum preparation

The fungal strains were cultured, and inoculum was prepared as described by Schweiger et al. (2021). Two genotypes of the *T. virens* strain Gv29.8 were used in the study: the wild-type and the *Δvir4* knockout mutant, which is defective in sesquiterpene biosynthesis, hereinafter referred to as “wild-type” and “mutant”, respectively. Both fungal strains were propagated on potato dextrose agar (PDA) (Carl Roth GmbH, Karlsruhe, Germany). Regular sub-culturing was done for four successive generations from the stock culture in sterile Petri dishes containing PDA, and the plates were kept in an environmental chamber under controlled conditions of 25 ± 2 °C; 65% RH; 12: 12 h light: dark for 7 days to promote conidiation. To harvest the conidia, fungal plates were flooded with 15–20 ml of sterile water using a pipette. A microscope slide was used to gently scrape the surface of the media plate to detach the conidia from the mycelium. Using a funnel, the resulting suspension was then filtered through a cheesecloth into an Erlenmeyer flask. The final concentration of conidia for each fungal strain was adjusted to 1 × 10^6^ conidia/ml to be used in subsequent inoculation procedures.

### Plant material and fungal inoculation

Seeds of maize, hybrid line 34H31 (Pioneer^®^ Brand Products, Gisborne, New Zealand), were used in the experiments, and the inoculation procedure followed Nogueira-Lopez et al. (2018). Prior to inoculation, the seeds were surface sterilized by submerging them in 2% sodium hypochlorite (NaOCl) solution, followed by 70% ethanol for 7 min each. The sterilization process was followed by five washes with autoclaved tap water. The success of sterilization was confirmed by plating 100 µL of water from the final rinse on PDA (Carl Roth GmbH, Karlsruhe, Germany) and incubating under the same conditions used for fungal culture preparation. Seeds were placed in Petri dishes with moistened, sterilized filter paper to pre-germinate overnight. The following day, seeds were inoculated with the fungal spore suspension individually at a concentration of 1 × 10^6^ conidia/seed under a laminar flow hood using a pipette. Three treatment groups were prepared: (1) seeds inoculated with *T. virens* wild-type strain, (2) seeds inoculated with the mutant strain, and (3) a control group where kernels were mock-inoculated with sterilized water using the same method as the fungal spore treatment. All procedures were conducted under sterile conditions to prevent contamination. Treated seeds were sown in plastic pots (10 cm diameter × 10 cm height) containing gamma-irradiated soil (1:4, sand: potting mix Fruhstorfer Erde Typ P; Hawita Gruppe GmbH, Werk Lauterbach, Germany). The pots were maintained in climate-controlled chambers with 25 °C; 80% RH; 16:8 h light: dark to allow for proper growth of the maize plants.

### Herbivore performance bioassay

To assess the impact of fungal colonization on herbivore survival and weight gain, 2nd instar larvae of *H. armigera* were weighed and randomly assigned to maize seedlings at BBCH stage 12 (mostly two leaves unfolded, 7 days old). Each seedling received a single larva placed into the whorl. The total sample size was 24 seedlings per treatment group (control, wild-type, mutant). To prevent larval escape and ensure airflow, each potted seedling was covered with a perforated cellophane plastic bag. The plants were then placed in an environmental chamber (25 °C, 80% RH, 16:8 h light: dark). The caterpillars were weighed again 3, 6, and 9 days post infestation (dpi) to determine the weight gain across different treatments. Additionally, survival was monitored daily for each treatment group throughout the 9-day feeding period to track mortality rates.

### Collection and analyses of the volatile blend

To collect and analyze the VOCs emitted by maize seedlings treated with *T. virens* wild-type, mutant, or control, a dynamic headspace VOCs collection system was employed, consisting of the bottom shelf of a six-arm olfactometer (as described by Turlings et al. 2004). Maize seedlings were infested with ten 2nd instar larvae of *H. armigera* one day before volatile collection. Individual seedlings were placed in one of the glass vessels of the olfactometer. The VOCs were collected using volatile trapping filters containing 30 mg of 80–100 mesh Porapak Q (Volatile Collection Trap LLC, FL, USA), connected to the outlet of the odor source vessel. A central in-house compressor provided humidified and filtered air at an inflow rate of 1 l/min. Air was pulled out from the odor source vessels at a flow rate of 0.8 l/min, using a membrane pump. The VOCs collection started daily at 10:00 a.m. and lasted for four hours. All equipment, including glassware and vessels, was thoroughly cleaned with demineralized water, 99.5% acetone, and oven-dried at 180 °C for two hours to prevent contamination. The filters were washed with 1 ml of dichloromethane (DCM) before use. After VOCs collection, the trapped VOCs were eluted from the filters by rinsing them with 150 µl of DCM into 1 ml glass vials. The samples were stored at −80 °C for further analysis, with 200 ng of 1,2,3,4-tetrahydronaphthalene added as an internal standard. For analysis, 40 µl of the final solution was transferred to glass vials with glass inserts for the GC-MS autosampler. A 2 µl aliquot was injected in pulsed splitless mode into the GC-MS system (5977B HES MSD, Agilent Technologies). The GC oven temperature was initially set at 40 °C for 3 min, then increased gradually at a rate of 8 °C min⁻¹ to 320 °C, which was held for 10 min. Helium was used as the carrier gas at a constant flux of 1.5 ml/min. Chromatograms were automatically integrated using Agilent MSD ChemStation software and mass spectra were compared to those in the libraries NIST17 and Wiley11 for tentative identification. For further confirmation of compound identities, the retention index (RI) of each compound was calculated and compared to the RI values listed in the Van Den Dool and Kratz RI Table available in the NIST Chemistry Webbook library. Quantification of the compounds was based on their mass peak areas relative to the peak area of the internal standard. This method allowed for relative comparisons of the amounts of each volatile emitted in the treatments. The experimental treatments consisted of the following setups with each treatment placed in one of the glass cylinders of the olfactometer: (1) maize, (2) maize infested with herbivores, (3) maize colonized by the wild-type and infested with herbivores, (4) maize colonized by the mutant and infested with herbivores, along with (5) a blank (empty collection vessel to control for background contamination).

### Response of M. pygmaeus to Herbivore-Induced Maize Volatiles

Y-tube olfactometer assays were conducted to assess the response of *M. pygmaeus* to volatiles emitted by maize treated with fungi and/or herbivores. The Y-tube had an 8 mm inner diameter, a 20 cm length for both the entry and side arms, and a 70° angle between the side arms. The methods were adapted from Lins et al. (2014), Maselou et al. (2019), and Silva et al. (2021). Maize seedlings (treated with fungal strains or left untreated as control) were infested with ten 2nd instar *H. armigera* larvae one day before the experiment to induce the release of VOCs. Seedlings were enclosed in polyethylene bags without plasticizers (Toppits^®^ Bratschlauch; Cofresco, Melitta Group, Germany) sealed at both ends. Two tubes (Tygon E-3603; Saint-Gobain Performance Plastics France) were inserted into the bags: one to allow inlet air from the air pump and the other to connect to the arms of the Y-tube olfactometer. Humidified and charcoal-filtered air was provided by an air compressor (Stimulus Controller CS-55 V2, Ockenfels Syntech GmbH, Germany), with the airflow adjusted to 0.3 l min⁻¹. Adults of *M. pygmaeus* were starved for 18 h before the experiment. Each individual insect was introduced into the central arm of the olfactometer and observed for a maximum of 10 min to make a choice by moving into one of the arms connected to an odor source. Insects moving 10 cm past the branching point towards one of the odor sources were considered to have made a choice. Insects that did not select a side within the 10-min period were classified as unresponsive and were excluded from the analysis. Five adult female insects were tested per plant set, and after each test, the olfactometer and odor sources were replaced. The control treatment followed the same procedure but without herbivore infestation. Before use, all olfactometer components were cleaned with Contrad 70^®^ detergent (Decon Laboratories Limited, VWR International, Hove, East Sussex, UK), rinsed with distilled water, and air-dried overnight at room temperature. Experiments were conducted at room temperature under a fume hood, and to avoid positional bias, the positions of the odor sources (infested vs. healthy) were alternated between the left and right side arms.

To compare the preferences of *M. pygmaeus* for different volatile profiles, six treatment combinations were tested pairwise using the Y-tube olfactometer. The treatment comparisons were as follows: (1) maize vs. maize infested with herbivores (M vs. MH); (2) maize vs. maize inoculated with the wild-type (M vs. MW); (3) maize vs. maize inoculated with the *vir4* mutant (M vs. MV); (4) maize infested with herbivores vs. maize inoculated with wild-type and infested with herbivores (MH vs. MWH); (5) maize infested with herbivores vs. maize inoculated with mutant and infested with herbivores (MH vs. MVH) (6) maize inoculated with wild-type and infested with herbivores vs. maize inoculated with mutant and infested with herbivores (MWH vs. MVH). Individuals of *M. pygmaeus* were tested only once, with a total of 30 insects per treatment pair. The positions of the odor sources connected to the olfactometer were exchanged after testing five insects, and seedlings were discarded after each test.

### Data analyses and statistical procedures

All statistical analyses and data visualizations were performed using R. A linear mixed-effects model was fitted to assess the effects of treatment, time, and their interaction on caterpillar weight gain. Treatment and time were treated as fixed effects, while individual caterpillars were considered random effects to account for repeated measures. Tukey’s Honestly Significant Difference (HSD) test, adjusted for multiple comparisons, was used for post-hoc mean comparisons. These analyses were conducted using the *lme4*, *car*, and *emmeans* packages in R. The relevant statistical assumptions were thoroughly evaluated. Normality of residuals was assessed through graphical inspections and the Shapiro-Wilk test, while homoscedasticity was evaluated using Levene’s test. Kaplan-Meier survival analysis, along with the log-rank test, was used to determine statistical differences in caterpillar survival across treatments. For the analysis of volatile emissions, the total volatile emission data were log-transformed to meet the assumptions of ANOVA and Tukey’s HSD test was conducted for mean separation. The quantities of individual VOCs among treatments were compared using the Kruskal-Wallis non-parametric test followed by Dunn test with a Bonferroni correction. Heatmap was generated using the *pheatmap* package in R and color gradients were customized using the *RColorBrewer* package. Prior to principal component analysis (PCA), VOC data were log-transformed to reduce skewness and stabilize variance among variables. The analysis was then performed on the log-transformed, mean-centered and scaled variables. Data processing and visualization were done with the packages *dplyr* and *ggplot2*, including 95% confidence ellipses to illustrate group separation. To test the null hypothesis of no preference by the predator between the two odor sources, a Chi-squared (χ²) test was performed. A significance threshold of *p* ≤ 0.05 was applied for all statistical tests.

## Results

### Herbivore performance and survival

**Fig. 1:**
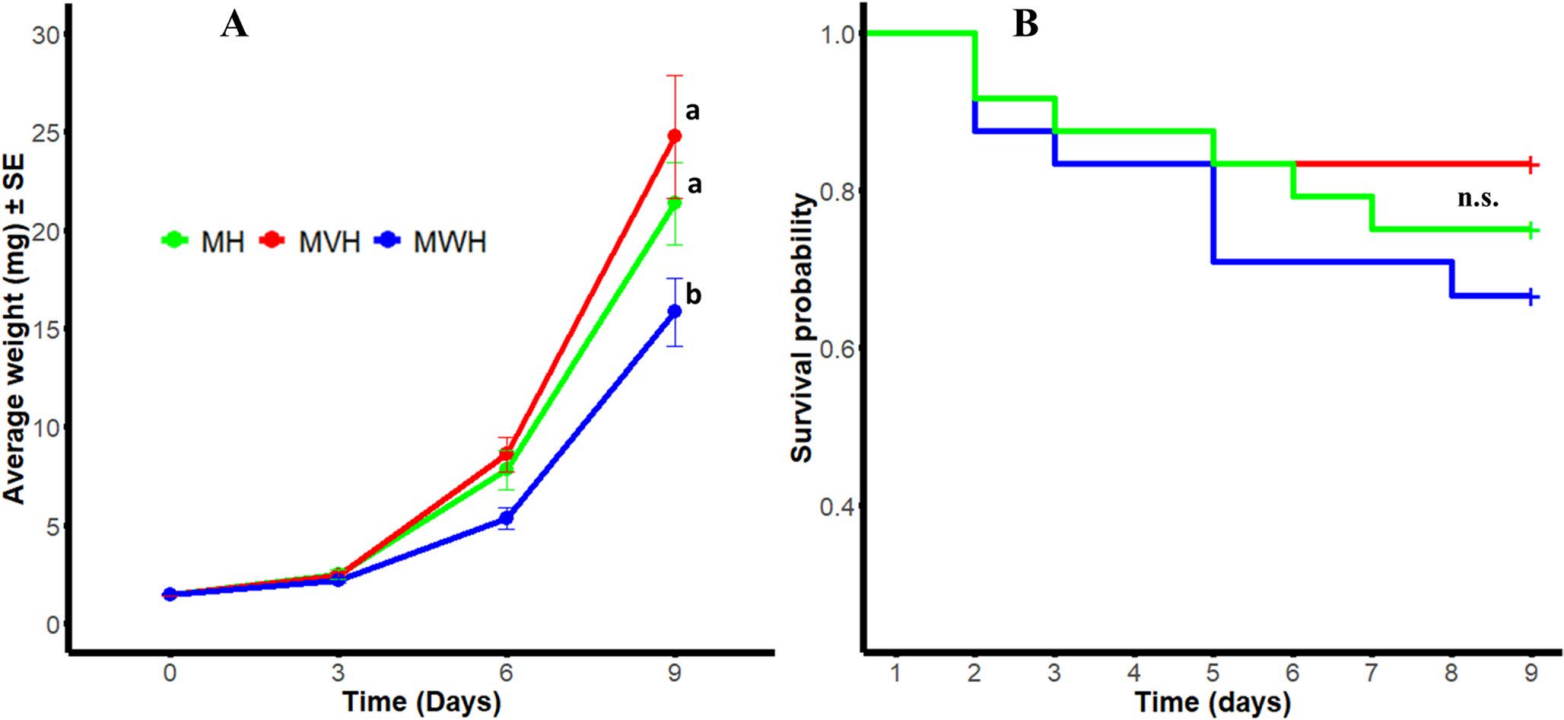
Performance of *Helicoverpa armigera* on maize seedlings colonized by fungal strains. (**A**) Caterpillar weight gain over time since infestation. The Y-axis represents caterpillar weight, while the X-axis shows days after feeding. Data were analyzed using a linear mixed-effects model, followed by Tukey’s HSD test (*p* ≤ 0.05) for mean separation. Different letters above the lines indicate significant differences among treatments. (**B**) Survival rate of *H. armigera* caterpillars over the 9-day experimental period. Kaplan-Meier survival curves with log-rank test (*p* ≤ 0.05) showed no significant differences among treatments (*p* > 0.05). Data for surviving caterpillars were censored after day 9. n.s.= not significant. Treatments: MH = non-colonized maize infested with *H. armigera* caterpillars (control), MVH = maize inoculated with the vir4 mutant and infested with caterpillars, MWH = maize inoculated with wild-type *T. virens* and infested with caterpillars (*n* = 24)

Larval performance was evaluated for each treatment by monitoring weight gain at various time points post-feeding. Statistically significant differences in fresh body mass were observed among caterpillars fed on different treatments (Fig. 1A). A linear mixed-effects model revealed significant main effects for both treatment (F_2.64, 11_ = 3.717, *p* = 0.02) and day (F_3, 177.31_ = 159.68, *p* < 0.001), as well as a significant interaction between treatment and day (F_6, 177.31_ = 3.026, *p* = 0.007). Further pairwise comparisons showed that by day 9, caterpillars feeding on maize seedlings colonized by the wild-type genotype had significantly lower body weights compared to those on both the control (*p* = 0.01) and mutant (*p* < 0.001) treatments (Fig. 1A). On the other hand, no significant differences in caterpillar survival were observed among the treatments over the 9-days experimental period, the minor early mortality occurred in some treatments including control may potentially be due to handling stress or natural causes (Fig. 1B) (Log-Rank test, χ² = 1.63, df = 2, *P* = 0.44). The experiments were terminated on day 9, as some seedlings in certain treatments (i.e., control or mutant) were fully consumed by the caterpillars towards the 9th day.

### VOCs emission

**Fig. 2:**
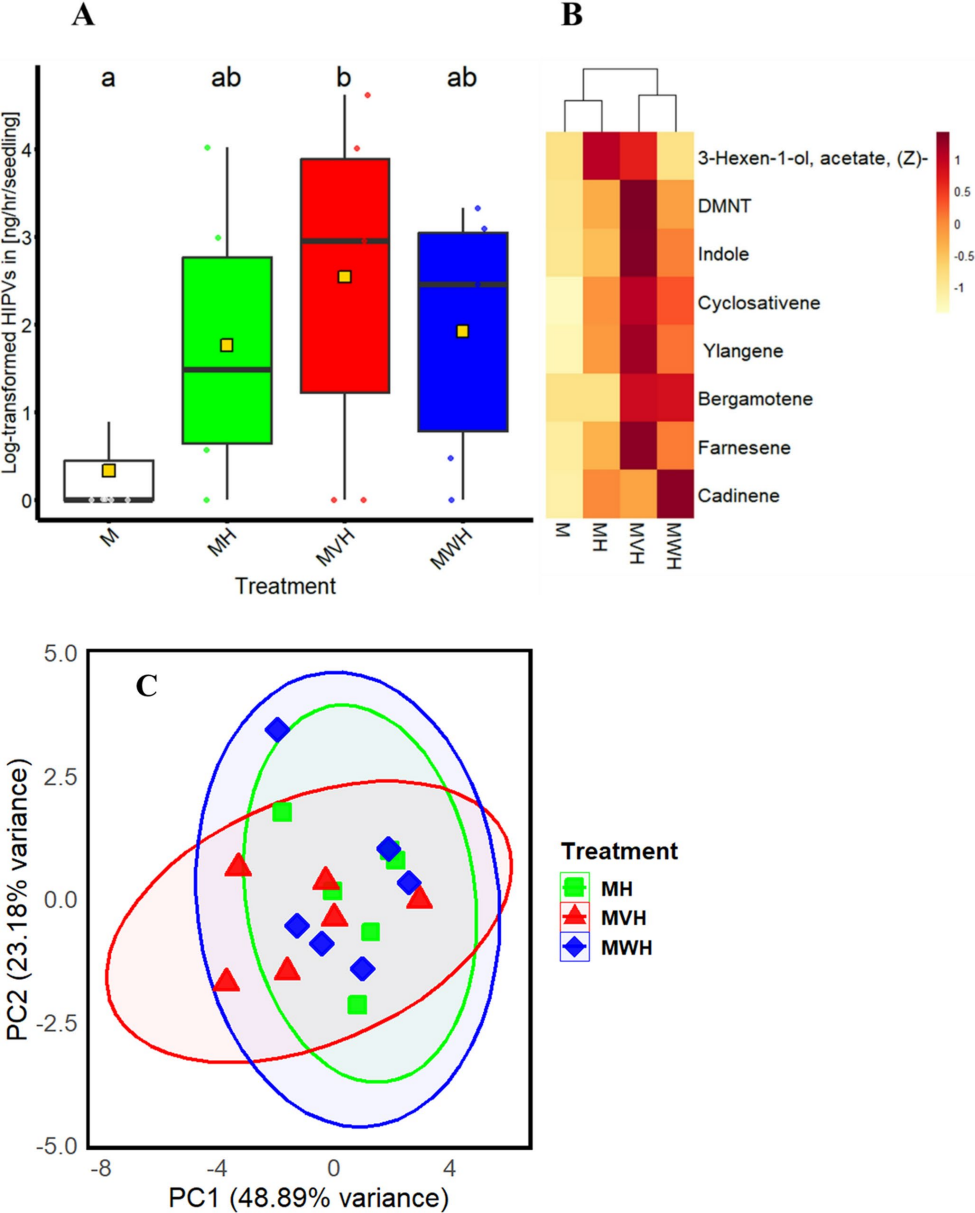
Volatile organic compounds profile of maize seedlings subjected to different treatments. (**A**) Box-whisker plots displaying log-transformed data on total volatile emission for each treatment. Medians are shown as horizontal lines, interquartile ranges (IQR) are indicated by boxes, whiskers extend to 1.5 times the IQR, and outliers are represented by dots outside the whiskers. The mean for each treatment is marked by a yellow square with black outline. Different letters denote statistically significant differences between treatments as determined by ANOVA and Tukey’ at *p* ≤ 0. 05. (**B**) Heatmap illustrating the abundance and clustering of VOCs across various treatments. The color intensity represents the relative concentration of each compound. Treatments include: M = uninfested control maize, MH = maize infested with *Helicoverpa armigera* caterpillars, MVH = maize inoculated with mutant and infested with caterpillars, and MWH = maize inoculated with wild-type and infested with caterpillars. (**C**) Principal Component Analysis score plot showing the variance explained by the first two principal components (PC1 and PC2), which account for 48.89% and 23.18% of the total variance, respectively

Herbivory induced a distinct volatile bouquet, with some qualitative differences attributed to the fungal strains. Comparing the wild-type and mutant treatments, certain compounds were detected in one treatment but absent in the other. For instance, (*Z*)−3-hexen-1-yl acetate was not found in the wild-type treatment but was present in the mutant treatment. Additionally, α‑bergamotene was identified in both fungal treatments but was absent in the herbivore-only treatment (Table 1). However, the quantity of individual VOCs did not differ statistically among treatments. ANOVA revealed significant differences in total volatile emissions between treatments (F_3, 24_ = 3.18, *p* = 0.04; Fig. 2A). A Tukey post-hoc test indicated that healthy maize emitted significantly fewer volatiles compared to the mutant-inoculated treatment (*p* = 0.03), while differences with other treatments were not statistically significant. Only trace amounts of VOCs were released from control maize. A heatmap visualization (Fig. 2B) illustrates the variation in volatile profiles across treatments, indicating that herbivory had the strongest effect on VOC emission patterns, whereas fungal colonization (wild type or mutant) caused only minor quantitative variation. Consistently, the principal component analysis (PCA) (Fig. 2C) did not show a clear separation between the treatments, supporting the conclusion that overall VOC blends were not markedly distinct. PC1 and PC2 together explained 72.16% of the total variance, reflecting overlapping yet subtly modulated VOC emission patterns between the mutant- and wild-type-colonized plants. Untreated maize samples were not included in the analysis as most compounds were absent.

**Table 1:** Estimated total VOC emission rate (ng/h/plant) from maize seedlings under different treatments: M = uninfested control maize, MH = maize infested with *Helicoverpa armigera* caterpillars, MVH = maize inoculated with the mutant and infested with caterpillars, and MWH = maize inoculated with the wild-type and infested with caterpillars. Single VOCs were compared among treatments using the Kruskal-Wallis non-parametric test. Values are presented as mean ± standard error (SE), *n* = 7. Value in the same row sharing the same letters are not statistically different at *p* ≤ 0.05. n.d.= not detected

Compound	Total VOCs emission ng/h/plant ± SE				*p* value
	M	MH	MVH	MWH	
(Z)−3-Hexen-1-yl acetate	n.d.	5.97 ± 5.97^a^	4.74 ± 2.27^a^	n.d.	> 0.05
DMNT	n.d.	3.33 ± 1.36 ^a^	12.71 ± 5.10^a^	4.00 ± 1.70 ^a^	> 0.05
Indole	n.d.	2.26 ± 0.82 ^a^	11.12 ± 6.19 ^a^	4.90 ± 1.70 ^a^	> 0.05
Cyclosativene	0.37 ± 0.25 ^a^	1.02 ± 0.44 ^a^	1.60 ± 0.54 ^a^	1.23 ± 0.60 ^a^	> 0.05
Ylangene	0.26 ± 0.19^a^	0.60 ± 0.33^a^	1.02 ± 0.33^a^	0.69 ± 0.38^a^	> 0.05
α-Bergamotene	n.d.	n.d.	0.08 ± 0.08^a^	0.83 ± 0.08^a^	> 0.05
β-Farnesene	n.d.	0.21 ± 0.11^a^	0.74 ± 0.33^a^	0.36 ± 0.17^a^	> 0.05
δ-Cadinene	n.d.	0.06 ± 0.06^a^	0.05 ± 0.05^a^	0.14 ± 0.14^a^	> 0.05

### Olfactometer response of the predator

**Fig. 3:**
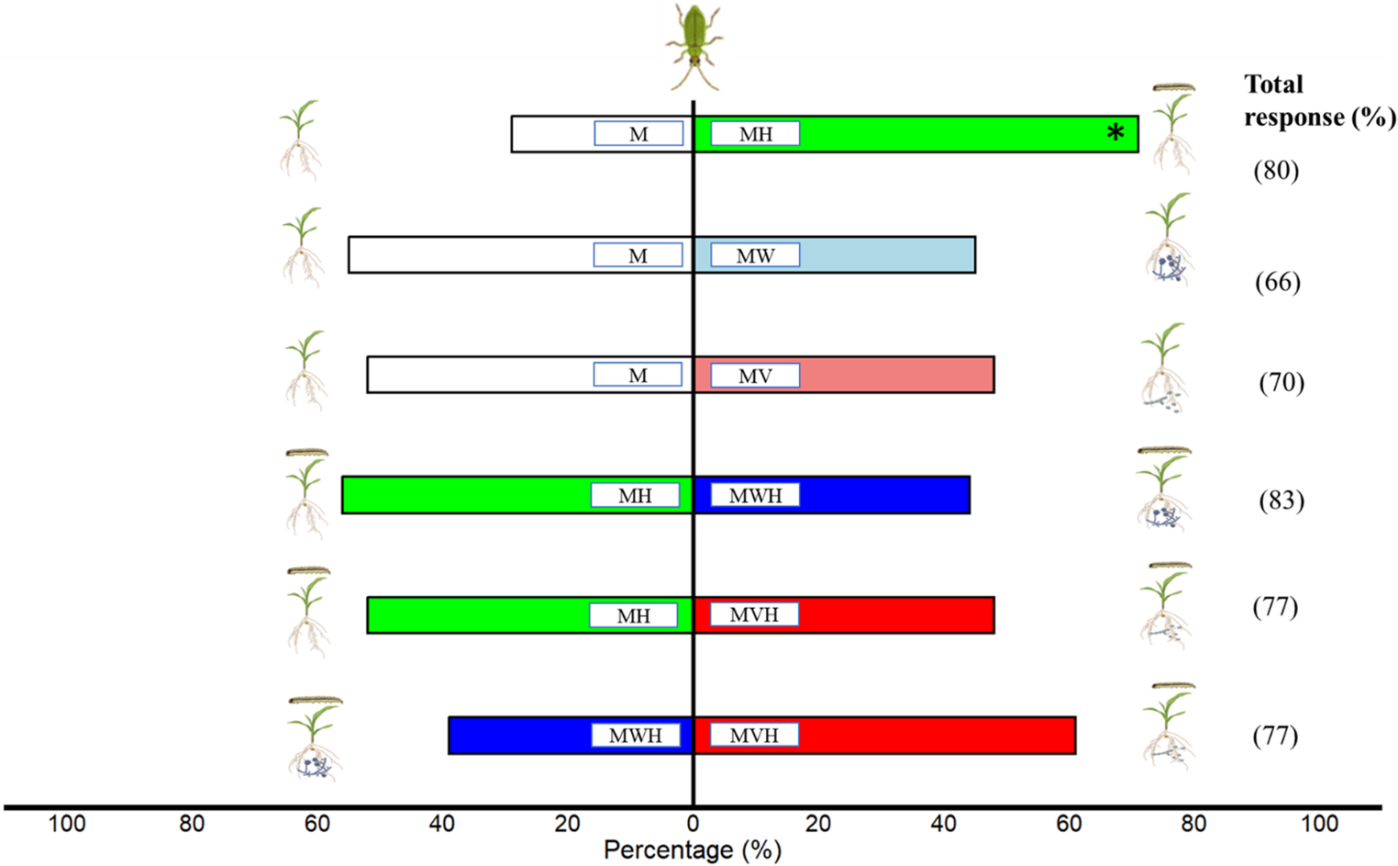
Response of *Macrolophus pygmaeus* to maize volatiles in a Y-tube olfactometer. The bars represent the percentage of responding insects (*n* = 30) for different treatment combinations: M = uninfested control maize, MH = maize infested with *Helicoverpa armigera* caterpillars, MV = maize inoculated with mutant, MVH = mutant-inoculated maize infested with caterpillars, MW = maize inoculated with wild type, and MWH = wild-type-inoculated maize infested with caterpillars. Predator response rates are shown as percentages on the right side of the plot. Pairwise comparisons between treatments were performed using Chi-squared (χ²) tests to assess predator preference, with significant differences (*p* ≤ 0.05) indicated by asterisks (*)

The response of *M. pygmaeus* to volatile blends released by maize seedlings colonized by different fungal strains, with or without herbivory, was assessed using a Y-tube olfactometer. The choices made by the predator, represented as percentages, indicate their preference for one arm of the olfactometer over the other (Fig. 3). The predators significantly preferred herbivore-damaged maize seedlings over healthy seedlings when given a choice between healthy and damaged seedlings (χ² = 4.17, df = 1, *P* = 0.04) (Fig. 3). However, Chi-squared tests indicated no significant preference for volatile blends associated with different fungal treatments used in different combinations. Specifically, predator preference did not differ significantly between maize inoculated with the wild-type and infested with caterpillars (MWH) and maize inoculated with the mutant and infested with caterpillars (MVH) (χ² = 1.09, df = 1, *P* = 0.29). Similarly, no significant differences were observed between uninfested control maize (M) and wild-type-inoculated maize (MW) (χ² = 0.20, df = 1, *P* = 0.65); uninfested control maize (M) and mutant-inoculated maize (MV) (χ² = 0.40, df = 1, *P* = 0.82); herbivore-infested maize (MH) and wild-type-inoculated, herbivore-infested maize (MWH) (χ² = 0.36, df = 1, *P* = 0.54); or herbivore-infested maize (MH) and mutant-inoculated, herbivore-infested maize (MVH) (χ² = 0.04, df = 1, *P* = 0.83).

## Discussion

*Trichoderma* spp. are widely recognized as effective biocontrol agents against phytopathogens, yet their role in mediating plant-insect interactions remains underexplored (Poveda 2021; Lelio et al. 2023). Building on the findings of Schweiger et al. (2021), which demonstrated that colonization of maize roots by wild-type and mutant *T. virens* differentially alters the plant root metabolome, this study investigated whether these metabolic changes influence the development and survival of *H. armigera* caterpillars aboveground. Additionally, we assessed the effects of *T. virens* colonization on indirect defense mechanisms involving the predator *M. pygmaeus*. Our results demonstrate that maize colonized by wild-type *T. virens* significantly inhibited *H. armigera* caterpillar development compared to mutant-colonized and control plants, suggesting that *T. virens* enhances direct systemic resistance in maize. This effect is likely mediated by fungal sesquiterpenes encoded by the *vir4* gene cluster, underscoring their importance in plant defense.

Induced systemic resistance is a key defense mechanism in plants, wherein beneficial rhizosphere microbes, such as *Trichoderma* spp., prime plants for enhanced defense against pathogens and herbivores (Pieterse et al. 2014). *Trichoderma* spp. produce a diverse array of bioactive secondary metabolites, including sesquiterpenes, which play critical roles in plant-microbe and plant-insect interactions (Kramer and Abraham 2012; Vinale et al. 2008; Reino et al. 2007). These metabolites act as signaling molecules and priming agents, systemically enhancing plant defense responses when released into the rhizosphere (Contreras-Cornejo et al. 2016; Shoresh et al. 2010; Pieterse et al. 2014). The reduced performance of *H. armigera* on maize colonized by wild-type *T. virens* may thus be attributed to the priming or induction of plant defenses by sesquiterpenes encoded within the *vir4* gene cluster. Indeed, Padilla-Arizmendi (2019) reported that colonization of maize roots by wild-type and mutant *T. virens* results in distinct metabolic profiles in the leaves, supporting the hypothesis that *T. virens* influences systemic plant metabolism. Nevertheless, further research is needed to identify the specific bioactive metabolites responsible for reducing herbivore performance. This finding ist consistent with previous studies demonstrating *Trichoderma*-mediated defense priming in various crops against herbivores from different feeding guilds (Alınç et al. 2021; Coppola et al. 2019b; Morán-Diez et al. 2021; Van Hee et al. 2025). Moreover, a direct deterrent effect of *vir4*-derived metabolites that are potentially translocated from roots to shoots cannot be excluded and warrants further investigation.

Our findings are aligned with previous research on *Trichoderma*-plant-insect interactions. For instance, *T. gamsii* altered the foliar metabolome of *Arabidopsis thaliana*, reducing weight gain in *Trichoplusia ni* (Zhou et al. 2018). Similarly, *T. atroviride* colonization limited herbivory by *Spodoptera frugiperda* through the induction of plant resistance, which was partly mediated by fungal-derived antifeedant secondary metabolites (Contreras-Cornejo et al. 2018). It also negatively impacted the development of *S. littoralis* and aphids via transcriptional changes in defense-related genes (Coppola et al. 2019b). Inoculation with *T. harzianum* reduced the growth rate of *Nezara viridula* by enhancing direct defense mechanisms and priming tomato plants (Alınç et al. 2021) while also enhancing maize root resistance to *Phyllophaga vetula* through the antifeedant activity of fungal-derived 6-pentyl-2 H-pyran-2-one (6-PP) (Contreras-Cornejo et al. 2021). Additionally, *T. afroharzianum* and *T. atroviride* have been shown to enhance tomato resistance against *S. littoralis* and *M. euphorbiae* (Lelio et al. 2021, 2023). Collectively, these findings underscore the potential of *T. virens* in enhancing maize direct systemic resistance against *H. armigera*. Although no direct mortality of *H. armigera* caterpillars was observed in our study, the relatively short experimental period (9 days) may have limited the detection of longer-term effects.

Maize responds to herbivory by releasing a blend of volatiles that attract natural enemies and prime neighboring plants for defense (Turlings and Ton 2006; Kim and Felton 2013; Thompson et al. 2023). Beneficial fungal colonization can influence HIPV emissions (Holopainen and Gershenzon 2010; Pangesti et al. 2013; Fernandez-Conradi et al. 2018), potentially shaping interactions at higher trophic levels. We investigated whether *T. virens* colonization alters maize HIPV emissions induced by *H. armigera* feeding. Our study identified a range of volatiles, including monoterpenes, sesquiterpenes, aromatic compounds, and green leaf volatiles (GLVs), primarily released in response to herbivory. However, *T. virens* root colonization had little influence on the composition of HIPVs emitted by maize leaves. Despite some variability in individual compounds such as (*Z*)−3-hexen-1-ol acetate, neither the wild-type nor the *vir4* mutant significantly affected the overall HIPV blend.

In contrast, fungal-induced changes in VOC emissions and indirect plant defenses has been documented in other systems. For example, *T. harzianum* enhanced methyl salicylate and β-caryophyllene emissions in aphid infested tomatoes, increasing aphid parasitoid attraction (Coppola et al. 2017). Similarly, *T. harzianum* and *T. atroviride* improved maize resistance to herbivores by modifying volatile terpene emissions that attract predators (Contreras-Cornejo et al. 2018, 2021). The fungus *T. atroviride* also influenced *S*. *littoralis* and *M. euphorbiae* survival and growth rates via transcriptional changes in defense genes while enhancing parasitoid attraction through altered VOC profiles in tomato, even in the absence of herbivory (Coppola et al. 2019a). Additionally, *Fusarium solani* strain K reduced *Tetranychus urticae* performance in tomato by modifying gene expression and VOC emissions, enhancing *M. pygmaeus* attraction (Pappas et al. 2018). Similarly, *T. longibrachiatum* increased *M. pygmaeus* attraction through HIPV modulation (Battaglia et al. 2013). However, in our study, *T. virens* colonization did not significantly alter maize VOC profiles, suggesting that the *Trichoderma* effects on tri-trophic interactions may be strain- and host-specific (Rodriguez and Redman 2008). Different *Trichoderma* species and strains exhibit substantial variation in the production of specialized metabolites, including VOCs that can serve as signaling molecules for host plants. Consequently, plant responses to *Trichoderma* colonization can differ markedly among host-microbe combinations as shown for example in *Arabidopsis thaliana* (Nieto-Jacobo et al. 2017; van Zijll de Jong et al. 2023).

In Y-tube olfactometer assays, *M. pygmaeus* exhibited a significant preference for maize HIPVs induced by *H. armigera* feeding, but fungal colonization did not further influence predator attraction to maize colonized by the fungal strains. This suggests that *T. virens* does not markedly alter VOC profiles in a way that affects third-trophic interactions. Similar findings were reported for *T. harzianum*, which also did not significantly modify tomato HIPVs or influence *M. pygmaeus* attraction (Meesters et al. 2024). Equally, fungal-derived volatiles did not affect the indirect defense of *Brassica rapa* against *Pieris brassicae* when plant roots were directly exposed to fungal VOCs, nor did they influence parasitism by *Cotesia glomerata* or alter the amount or quality of plant-emitted volatiles, resulting in no effects on indirect defense (Moisan et al. 2020).

In conclusion, this study demonstrates that *T. virens* modulates plant-herbivore interactions but does not significantly affect plant-herbivore-predator dynamics. Our findings highlight the crucial role of fungal sesquiterpenes in enhancing maize resistance against *H. armigera* through direct defense mechanisms. To our knowledge, this is the first study to show that *T. virens* colonization negatively affects *H. armigera* performance via defense responses mediated by the *vir4* gene cluster. These results underscore the potential of *T. virens* in strengthening maize resistance to herbivory and emphasize the need for further research into the genetic and metabolic pathways underlying *T. virens*-induced direct defense against *H. armigera*.

## Supplementary Information

Below is the link to the electronic supplementary material.

ESM 1 (DOCX 1.83 MB)

## Data Availability

The authors declare that the data supporting the findings of this study are available within the paper. Should any raw data files be needed in another format they are available from the corresponding author upon reasonable request.

## Declarations

All authors have agreed upon the final version of the manuscript.
