# Supplementary material for: Sesquiterpene Biosynthetic Gene vir4 from Trichoderma virens Enhances Direct Herbivore Resistance while Maintaining Indirect Defense
Source: J Chem Ecol. 2026 Jan 13;52(1):4. doi: 10.1007/s10886-025-01681-4 (PMC12799740; doi:10.1007/s10886-025-01681-4)
Supplement: Supplementary file 1 — (DOCX 1.83 MB) [file 10886_2025_1681_MOESM1_ESM.docx]

# Supplementary Material


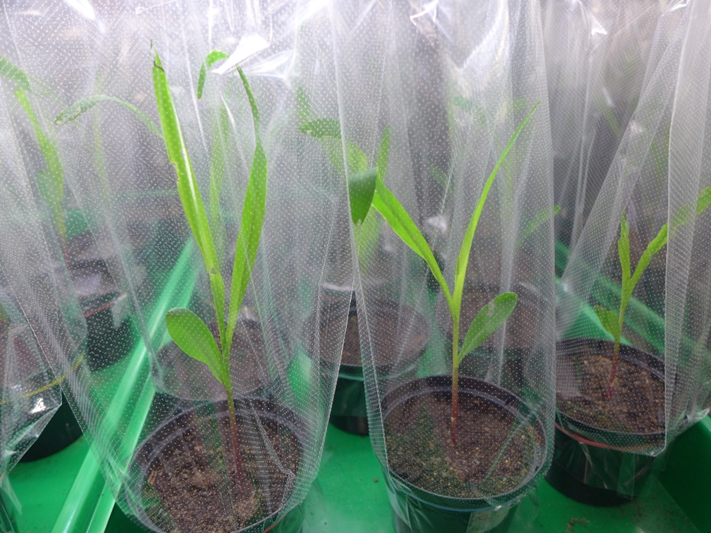


**Figure S1** Maize seedlings covered with perforated cellophane bags to test the effects *Trichoderma virens* root colonisation on the development of *Helicoverpa armigera.*
